# Paternity leave duration and maternal depressive symptoms: a longitudinal study of paternal involvement and coparenting in Japan

**DOI:** 10.3389/fpubh.2026.1891071

**Published:** 2026-07-22

**Authors:** Aiko Hironaka, Hiroko Watanabe

**Affiliations:** 1 Faculty of Health Science, Okayama University, Okayama, Japan; 2 Department of Children and Women’s Health, The University of Osaka Graduate School of Medicine, Osaka, Japan

**Keywords:** coparenting, maternal depressive symptoms, parity, paternal involvement, paternity leave, postpartum

## Abstract

**Background:**

Paternity leave may be associated with maternal mental health through fathers’ involvement and coparenting, but short-term family-process associations during the early postpartum period remain unclear.

**Objective:**

We examined modeled structural and indirect associations among paternity leave duration, mother-reported paternal involvement and coparenting, and maternal depressive symptoms from 3 to 6 months postpartum, separately by parity.

**Methods:**

This two-wave online survey included 396 Japanese mothers (244 primiparas and 152 multiparas). At the 6-month follow-up, mothers reported the father’s total paternity leave duration, categorized as no leave, short leave (1–31 days), or long leave (32 days or more). Paternal involvement at 3 and 6 months was modeled as a latent construct indicated by four domains of childcare and housework; coparenting and depressive symptoms were assessed using the CRS-J and CES-D, respectively. A multigroup two-wave structural equation model of structural and indirect associations was estimated. Indirect associations were evaluated using 5,000 bootstrap samples and bias-corrected 95% confidence intervals. All principal variables were mother-reported.

**Results:**

The freely estimated multigroup model showed good fit, χ^2^(194) = 309.282, CFI = 0.961, TLI = 0.946, and RMSEA = 0.039. Long leave, relative to no leave, was associated with greater paternal involvement at 3 months in both parity groups, whereas short leave was not clearly associated with paternal involvement. Paternal involvement and coparenting showed continuity from 3 to 6 months. The CRS-at-6-months to CES-D-at-6-months association and the total indirect association between long leave and CES-D were statistically supported within primiparas but not within multiparas. However, formal metric-invariant between-group comparisons were not significant for either the CRS-to-CES-D path, Δχ^2^(1) = 0.482, *p* = 0.488, or the total indirect association, ΔB = −0.225, 95% bias-corrected CI [−0.903, 0.441], *p* = 0.454.

**Conclusion:**

Long paternity leave was associated with greater mother-reported paternal involvement and short-term coparenting continuity. The modeled indirect association with maternal depressive symptoms was very small, and formal comparisons did not establish parity differences. These findings describe short-term observational associations rather than causal effects.

## 1 Introduction

Maternal depressive symptoms during the postpartum period do not only affect mothers’ personal well-being. Rather, these symptoms may also affect mothers’ relationships with their partners, mother–infant interaction, and child development (1). Accordingly, it is important to examine family processes that may be linked to mothers’ adjustment after childbirth. Although postpartum depressive symptoms have often been examined as an individual maternal outcome, recent studies have tended to focus on the family context surrounding mothers, including fathers’ participation in childcare and housework and how parents coordinate childrearing together (2, 3).

Paternity leave is relevant to maternal postpartum mental health because it may change what fathers are able to do at home. Time away from work can give fathers opportunities to care for the infant, help with housework, and support their spouses while new family routines are being formed. However, previous findings have been inconsistent regarding the influence of paternity leave on mothers’ mental health. Systematic review evidence suggests that more generous leave policies are often linked to improved maternal mental health (4). Meanwhile, individual studies have reported weaker or less consistent associations with mothers’ postpartum mental health (5, 6). Quasi-experimental evidence is also available. Persson and Rossin-Slater exploited a Swedish policy reform and found that greater paternal access to workplace flexibility during the postpartum period improved maternal health outcomes (7). This causal policy evidence is distinct from the present observational study, which examines how leave duration, paternal involvement, coparenting, and maternal depressive symptoms are patterned within families. Together, the available evidence suggests that associations between leave-taking and maternal mental health may depend partly on whether leave is accompanied by fathers’ day-to-day involvement in childcare and housework. Japan provides a distinctive context for examining paternity leave duration. Although fathers’ leave-taking has increased in Japan, the FY2024 Basic Survey of Gender Equality in Employment Management reported that 40.5% of fathers took childcare leave, including postnatal paternity leave, compared with 86.6% of women (8). Fathers’ leave also remains relatively short: in the FY2023 survey, more than half of male returnees from childcare leave had taken less than 1 month of leave (9). In addition, paternal childcare in Japan may be constrained by work-related time and workplace conditions; large Japanese studies have shown that longer paternal working hours are associated with lower engagement in multiple parenting behaviors and that work-related factors, including working and commuting hours, are linked to fathers’ childcare involvement regardless of mothers’ employment status (10, 11).

Previous studies have reported a similar pattern, while also leaving an important question unanswered. Knoester et al. (12) found that fathers who took paternity leave, and those who took longer leave, were more involved with their children over time. Petts and Knoester (13) used longitudinal data from the Fragile Families and Child Wellbeing Study to determine that fathers’ time off work after birth was related to better relationship and coparenting quality, partly through the involvement of the father. In Japan, Kasamatsu et al. (14) found that specific paternal childcare behaviors at 6 months were associated with lower maternal psychological distress at 1 year after delivery. These studies changed focus from leave-taking itself to what fathers do during and after leave, namely whether they become more involved in childcare and housework. However, less is known about whether these family processes are linked to maternal depressive symptoms over time. In particular, few studies have examined the continuity of paternal involvement and coparenting from 3 to 6 months postpartum or examined these pathways separately among primiparous and multiparous mothers in Japan.

The contribution of the present study is to jointly examine paternity leave duration, mother-reported paternal involvement, coparenting continuity, and maternal depressive symptoms within a single two-wave family-process model in Japan and to formally evaluate key apparent parity differences. This integrated model allows these processes to be examined together rather than as isolated pairwise associations. The 3–6-month interval captures a short early-postpartum period in which infant-care demands remain high and caregiving and coparenting routines continue to develop after the immediate post-birth period (15, 16). The analysis therefore addresses short-term continuity over this interval and does not assess persistence beyond 6 months.

Coparenting is one plausible family process linking paternal involvement in childcare and housework with maternal mental health. As defined by Feinberg (17), coparenting refers to how two adults raising children together coordinate their efforts by dividing caregiving tasks, backing each other up or not, and negotiating disagreements about parenting. This is conceptually distinct from couple relationship quality per se. Feinberg was explicit that coparenting pertains to the parenting relationship, not romantic closeness or general partnership satisfaction. This distinction is empirically important: coparenting quality has been more consistently tied to parenting-related outcomes than to general relationship functioning (17, 18). In Japan, Takeishi et al. (19) found that mothers who reported better coparenting experienced fewer depressive symptoms in the early postpartum period.

The temporal relationship between coparenting and parental depression is not well resolved. The two constructs are reliably correlated, but studies disagree on the causal relationship. Wells et al. (3) found evidence of influence in both directions: paternal depression predicted poor coparenting, and poor coparenting predicted increased depression. In contrast, Tissot et al. (20) argued that parental symptoms more often shape coparenting than the reverse. Studies of broader partnership quality also suggest the need for caution in proposing relationship directionality. Schwarze et al. (21) found that partnership quality and maternal depressive symptoms were strongly related from pregnancy to postpartum, but cross-lagged analyses did not identify a clear causal direction. However, partnership quality is not synonymous with coparenting, and their study did not examine paternity leave or fathers’ behavioral involvement. Thus, it remains unclear how fathers’ leave-taking and day-to-day involvement are linked to the continuity of coparenting and to maternal depressive symptoms during the postpartum months.

Parity requires separate consideration. Having a first child is a fundamentally different experience from adding to an established family. First-time parents must determine how to share caregiving for the first time while simultaneously navigating new demands on their time, their sense of self, and their relationship. This combination of unfamiliarity and adjustment may help explain the elevated stress, anxiety, and depressive symptoms documented among primiparous mothers and fathers during the early postpartum months (15).

Parity may shape early family processes, but any between-group differences require direct statistical testing. Primiparous families are establishing parenting roles, caregiving routines, and coparenting patterns for the first time during the transition to parenthood. Multiparous families may draw on pre-existing routines while simultaneously managing older-child care, more complex household logistics, and additional time demands. These different contexts provide a rationale for examining group-specific patterns, although the parity-stratified analyses were exploratory and were not assumed to demonstrate statistically significant differences between groups (15, 22, 23).

Against this background, the present study used a multigroup two-wave structural equation model to examine how paternity leave duration, mother-reported paternal involvement, coparenting continuity, and maternal depressive symptoms were associated from 3 to 6 months postpartum in Japan. The purpose was descriptive and mechanism-oriented rather than causal. We estimated the model separately for primiparous and multiparous mothers to describe group-specific patterns and, for the key apparent parity differences, formally tested whether the relevant coefficients differed between groups.

Accordingly, we hypothesized that:

*H*1: Long paternity leave, relative to no paternity leave, would be associated with greater paternal behavioral involvement in childcare and housework at 3 months postpartum, and paternal behavioral involvement would be associated with better coparenting at 3 months.

*H*2: Coparenting and paternal behavioral involvement in childcare and housework would show continuity from 3 to 6 months postpartum, with better coparenting at 6 months being associated with fewer maternal depressive symptoms at 6 months.

## 2 Materials and methods

### 2.1 Study design and participants

This study was a longitudinal follow-up of an online survey of Japanese mothers conducted at 3 months postpartum. The baseline survey was conducted from May to September 2025 through two recruitment channels: an online research company (Freeasy; iBRIDGE Corporation, Osaka, Japan) and an e-mail newsletter distributed by a baby-product company. Mothers were eligible if they were rearing a singleton infant, were able to complete the questionnaire in Japanese, and cohabited with the child’s father. Mothers with a history of mental illness were excluded.

**Figure 1:**
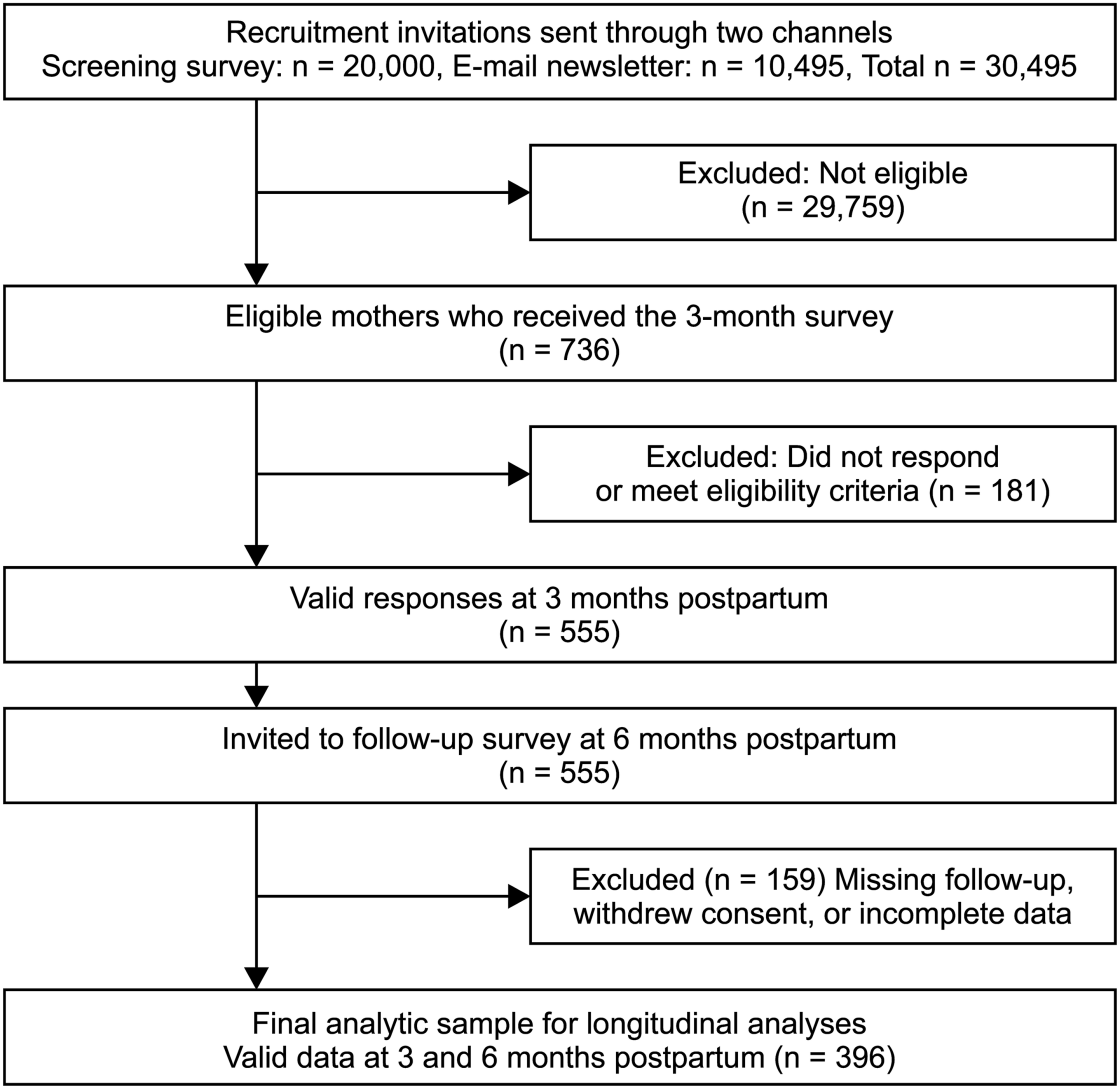
Flowchart for the study sample.

At baseline, 555 mothers provided valid responses at 3 months postpartum. These mothers were invited to participate in a follow-up survey 3 months later, when their infants were 6 months old. Of the mothers, 159 did not complete the follow-up survey, withdrew consent, or had missing or incomplete data for the focal variables. The final analytic sample consisted of 396 mothers, namely 244 primiparas and 152 multiparas. The participant recruitment and follow-up process is shown in Figure 1. Characteristics of the analytic sample are shown in Table 1.

**Table 1:** Characteristics of the analytic sample by parity.

Variable	Primiparas (*n* = 244)	Multiparas (*n* = 152)	*p* value
Maternal age	32.99 ± 4.68	33.63 ± 4.82	0.192†
Paternal age	34.74 ± 6.12	34.59 ± 5.78	0.801†
Perceived financial adequacy	2.57 ± 0.79	2.57 ± 0.79	0.986†
Maternal education, n (%)			0.403‡
High school or less	47 (19.3)	24 (15.8)	
Vocational school/junior college	48 (19.7)	25 (16.4)	
University or higher	149 (61.1)	103 (67.8)	
Paternal education, n (%)			0.120‡
High school or less	66 (27.0)	42 (27.6)	
Vocational school/junior college	40 (16.4)	14 (9.2)	
University or higher	138 (56.6)	96 (63.2)	
Economic status, n (%)			0.939‡
Very little financial leeway	28 (11.5)	16 (10.5)	
Limited financial leeway	66 (27.0)	45 (29.6)	
Moderate financial leeway	132 (54.1)	79 (52.0)	
Considerable financial leeway	18 (7.4)	12 (7.9)	
Maternal employment, employed, n (%)	174 (71.3)	113 (74.3)	0.511‡
Paternity leave duration, n (%)			0.447‡§
No leave	113 (46.3)	67 (44.1)	
Short leave (1–31 days)	57 (23.4)	44 (28.9)	
Long leave (32 days or more)	74 (30.3)	41 (27.0)	

Values are presented as mean ± standard deviation or n (%). Paternity leave duration represents the father’s total paternity leave duration reported at the 6-month follow-up. SD, standard deviation. † *p* values were calculated using independent-samples t tests. ‡ *p* values were calculated using chi-square tests. § The *p* value for paternity leave duration was for the comparison among the three primary leave categories: no leave, short leave, and long leave. Subcategories within short leave are descriptive only. Within the short leave group in the analytic sample (*n* = 101), the duration breakdown was: < 1 week (*n* = 8, 7.9%), 7–14 days (*n* = 24, 23.8%), 15–21 days (*n* = 11, 10.9%), and 22–31 days (*n* = 58, 57.4%).

### 2.2 Measures

Although the 6-month follow-up survey included the same set of measures as the baseline survey, the present longitudinal analysis focused on the variables included in the final structural model: fathers’ paternity leave duration, paternal behavioral involvement in childcare and housework, coparenting quality, and maternal depressive symptoms.

All principal study variables were reported by mothers. Mothers were selected as respondents because maternal depressive symptoms were the primary outcome and the study focused on paternal involvement and coparenting as family processes experienced and perceived by mothers. Accordingly, mother-reported paternal involvement and coparenting should not be interpreted as independent or objective assessments of fathers’ behavior or of the coparenting relationship.

#### 2.2.1 Paternity leave duration

At the 3-month assessment, mothers reported whether the child’s father had taken paternity leave and, if so, its duration at that time. At the 6-month follow-up, mothers reported the father’s total paternity leave duration. The primary SEM used the total duration reported at the 6-month follow-up, categorized as no leave (0 days), short leave (1–31 days), or long leave (32 days or more), consistent with the distribution and Japanese leave context (8, 9). For the attrition analysis, paternity leave duration reported at the 3-month assessment was used because 6-month follow-up data were unavailable for participants who did not complete the follow-up survey.

#### 2.2.2 Paternal behavioral involvement in childcare and housework

Paternal behavioral involvement in childcare and housework (hereafter, PI) was assessed using the Child Care Activities and Housework Scale (24). This instrument includes four subscales: Interactional Activities (e.g., talking to or holding the infant), Concrete Child Care Activities (e.g., bathing or changing clothes), Mental Support (e.g., listening to the mother’s concerns), and Housework (e.g., cooking, cleaning, and laundry). Items are rated on a 4-point Likert scale ranging from 1 (never do) to 4 (always do), with higher scores indicating more active paternal involvement. Mean scores were calculated for each subscale at both 3 and 6 months postpartum. In the present sample, Cronbach’s *α* coefficients were 0.768 for Interactional Activities, 0.839 for Concrete Child Care Activities, 0.758 for Mental Support, and 0.733 for Housework. In the present study, the four subscales were used as indicators of PI. In the longitudinal structural equation model (SEM), PI was modeled as a latent construct at 3 and 6 months, as indicated by the four observed subscale means. To account for indicator-specific stability across time, residual covariances were permitted between the corresponding 3-month and 6-month subscales.

#### 2.2.3 Coparenting relationship

Coparenting quality was assessed using the Japanese version of the Coparenting Relationship Scale [CRS; original version (25), Japanese version (26)]. The CRS consists of 35 items that are divided into seven subscales: Agreement, Closeness, Exposure to Conflict, Support, Undermining, Endorsement of Partner’s Parenting, and Division of Labor. Items are rated on a 7-point scale ranging from 0 to 6. Negative subscales, namely Exposure to Conflict and Undermining, are reverse-coded so that higher scores indicate better coparenting quality. In the present study, the CRS item-mean composite score was used at both 3 and 6 months postpartum. In the present sample, Cronbach’s *α* for the CRS item-mean composite score was 0.850.

#### 2.2.4 Maternal depressive symptoms

Maternal depressive symptoms were measured using the Japanese version of the Center for Epidemiologic Studies Depression Scale [CES-D; Japanese version (27), original version (28)]. The CES-D is a 20-item self-report measure that assesses the frequency of depressive symptoms in the prior week. Responses are rated on a 4-point Likert scale from 0 (rarely or none of the time) to 3 (most or all of the time). Four positively worded items are reverse-coded. Total scores range from 0 to 60, with higher scores indicating more severe depressive symptoms. In the present study, the CES-D total score was treated as a continuous variable at both 3 and 6 months postpartum. In the present sample, Cronbach’s *α* for the total score was 0.866.

#### 2.2.5 Covariates

Based on existing literature and theoretical relevance, maternal age, maternal employment status, and perceived financial adequacy were included as covariates. Maternal employment was treated as a binary variable (employed vs. not employed). Perceived financial adequacy was assessed in terms of four ordered categories, coded from 1 to 4, with higher scores indicating greater financial adequacy. As the primary aim of the study was to compare pathways between primiparous and multiparous mothers, parity was used as a grouping variable in the multigroup SEM rather than as a covariate. Descriptive statistics for the analytic sample are presented in Table 1.

### 2.3 Statistical analysis

Analyses were conducted using IBM SPSS Statistics version 30.0 and Amos version 30.0. Descriptive statistics and Pearson correlation coefficients were calculated separately by parity. Attrition analyses compared participants included in the final longitudinal analyses (*n* = 396) with those not included (*n* = 159). Independent-samples t tests were used for continuous baseline variables, and Pearson chi-square tests were used for categorical baseline variables. The attrition analysis used paternity leave duration reported at the 3-month assessment because 6-month paternity-leave data were unavailable for participants without follow-up data.

Before estimating the structural model, we evaluated the measurement model for paternal involvement using a multigroup longitudinal confirmatory factor analysis across parity groups. Paternal involvement at 3 and 6 months was specified as two correlated latent factors, each indicated by the four subscale mean scores. Residual covariances were specified between corresponding indicators across time. A configural model with freely estimated factor loadings was compared with a metric-invariance model in which the six freely estimated factor loadings were constrained to equality across groups. Model fit was evaluated using χ^2^, CFI, TLI, and RMSEA, together with changes in CFI and RMSEA and the chi-square difference test.

The primary analysis was a multigroup two-wave autoregressive structural equation model of structural and indirect associations estimated by maximum likelihood. Paternal involvement at 3 and 6 months was represented by latent factors, whereas CRS and CES-D were entered as observed composite scores. The model included stability paths from PI at 3 months to PI at 6 months, CRS at 3 months to CRS at 6 months, and CES-D at 3 months to CES-D at 6 months; structural associations from the leave-duration dummy variables to PI at 3 months, PI at 3 months to CRS at 3 months, PI at 6 months to CRS at 6 months, and CRS at 6 months to CES-D at 6 months; and covariate paths from maternal age, maternal employment, and perceived financial adequacy to PI at 3 months, CRS at 3 months, and CES-D at 6 months. The two leave-duration dummy variables were allowed to covary, and corresponding PI-indicator residuals were allowed to covary across time. The model focused on the hypothesized indirect pathways from paternity leave duration to CES-D at 6 months; no direct path between these variables was included. Accordingly, the reported total indirect association represents the sum of the model-specified indirect associations. Reciprocal cross-lagged paths among PI, CRS, and CES-D were not estimated.

Indirect associations were evaluated using 5,000 bootstrap samples and bias-corrected 95% confidence intervals. The freely estimated model was used to report group-specific standardized coefficients and R^2^ values. After metric invariance was established, formal between-group comparisons were conducted under metric-invariant structural models. The CRS-at-6-months to CES-D-at-6-months path was tested by comparing a freely estimated metric-invariant model with a nested model constraining that unstandardized path to equality. The difference between the parity-specific total indirect associations from long leave to CES-D was evaluated using a user-defined bootstrapped contrast. Model arrows represent model-specified associations and should not be interpreted as causal effects.

### 2.4 Ethical considerations

Before accessing the survey, participants were presented with an online information sheet describing the study and its ethical implications. Consent was indicated by checking a designated box, after which the questionnaire became accessible. The study protocol was reviewed and approved by the Ethics Committee of The University of Osaka Hospital (approval no. 24576).

## 3 Results

### 3.1 Participant characteristics

Of the 555 mothers who completed the baseline survey, 396 (244 primiparas and 152 multiparas) provided valid follow-up data and were included in the analyses. As shown in Table 1, the two groups were demographically comparable in terms of maternal and paternal age, parental education, economic status, employment, and leave distribution. Among primiparous families, 113 (46.3%) reported no paternity leave, 57 (23.4%) short leave, and 74 (30.3%) long leave. In the multiparous group the corresponding values were 67 (44.1%), 44 (28.9%), and 41 (27.0%).

Attrition analyses compared the 396 participants included in the final analyses with the 159 participants who were not included. The groups did not show clear differences in maternal age (*p* = 0.615), paternal age (*p* = 0.521), parity (*p* = 0.173), maternal employment (*p* = 0.828), perceived financial adequacy (*p* = 0.847), paternity leave duration reported at 3 months (*p* = 0.432), the four baseline PI domains (*p* values = 0.071–0.893), or baseline CES-D scores (*p* = 0.446). Participants included in the final analyses reported slightly higher baseline CRS total scores than those not included (3.01 ± 0.64 vs. 2.85 ± 0.64, *p* = 0.010, Cohen’s d = 0.24). Full results are presented in Supplementary Table S1.

### 3.2 Descriptive statistics, between-group comparisons, and bivariate correlations

Table 2 shows descriptive statistics and between-group comparisons for the principal study variables. No significant differences were observed between primiparous and multiparous mothers in PI, CRS, or CES-D scores at either 3 or 6 months postpartum.

**Table 2:** Descriptive statistics and between-group comparisons of principal study variables in primiparas and multiparas at 3 and 6 months postpartum.

Variable	Primiparas (*n* = 244)	Multiparas (*n* = 152)	*p* value
PI: Interactional activities, 3 months	3.12 ± 0.60	3.11 ± 0.56	0.975
PI: Interactional activities, 6 months	3.08 ± 0.55	3.08 ± 0.57	0.994
PI: Concrete child care activities, 3 months	2.87 ± 0.76	2.81 ± 0.72	0.469
PI: Concrete child care activities, 6 months	2.75 ± 0.71	2.73 ± 0.71	0.796
PI: Mental support, 3 months	3.00 ± 0.72	2.89 ± 0.75	0.143
PI: Mental support, 6 months	2.90 ± 0.67	2.85 ± 0.68	0.462
PI: Housework, 3 months	2.63 ± 0.76	2.55 ± 0.77	0.338
PI: Housework, 6 months	2.53 ± 0.77	2.44 ± 0.70	0.245
CRS total score, 3 months	3.05 ± 0.63	2.96 ± 0.65	0.182
CRS total score, 6 months	2.96 ± 0.65	2.92 ± 0.67	0.582
CES-D total score, 3 months	11.31 ± 8.89	11.75 ± 9.45	0.638
CES-D total score, 6 months	11.09 ± 8.75	12.13 ± 9.04	0.254

Values are presented as mean ± standard deviation. *p* values were calculated using independent-samples t tests. PI, paternal involvement in childcare and housework; CRS, Coparenting Relationship Scale; CES-D, Center for Epidemiologic Studies Depression Scale.

Bivariate correlations are shown separately for primiparas and multiparas in Supplementary Tables S3, S4. Within each parity group, the four PI domains at 3 months were moderately to strongly correlated and were positively associated with CRS at 3 months. PI domains at 3 months were also positively correlated with the corresponding PI domains at 6 months, and CRS at 3 months was strongly positively correlated with CRS at 6 months. In both groups, CES-D scores at 3 and 6 months were strongly positively correlated, whereas CRS at 6 months was negatively correlated with CES-D at 6 months. These correlations describe observed associations but do not verify temporal or causal ordering.

### 3.3 Multigroup structural equation model and structural associations

Before the structural model was evaluated, the multigroup longitudinal CFA showed excellent fit, χ^2^(30) = 33.849, *p* = 0.287, CFI = 0.998, TLI = 0.996, and RMSEA = 0.018, 90% CI [0.000, 0.044]. Standardized factor loadings ranged from 0.541 to 0.878, and all freely estimated loadings were significant (*p* < 0.001). The latent PI factors at 3 and 6 months correlated at 0.773 among primiparas and 0.715 among multiparas. Constraining the six freely estimated factor loadings to equality across parity groups did not significantly worsen model fit, χ^2^(36) = 44.467, *p* = 0.157; Δχ^2^(6) = 10.618, *p* = 0.101, ΔCFI = −0.002, and ΔRMSEA = 0.006, supporting metric invariance (Supplementary Table S7).

**Figure 2:**
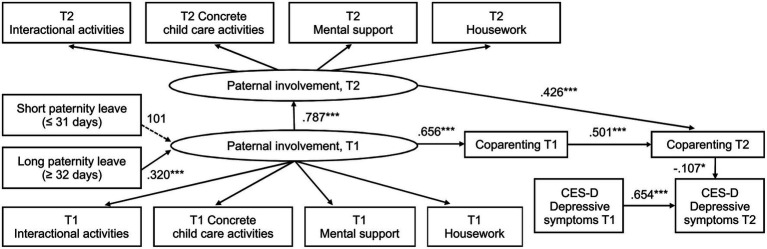
Two-wave structural equation model of modeled associations among paternity leave duration, paternal involvement, coparenting, and maternal depressive symptoms in primiparas. Note. Standardized path coefficients (*β*) from the freely estimated model are shown. Solid and dashed lines indicate paths whose 95% bias-corrected bootstrap confidence intervals excluded and included zero, respectively. T1, 3 months postpartum; T2, 6 months postpartum; PI, paternal involvement; CES-D, Center for Epidemiologic Studies Depression Scale. Short and long paternity leave were dummy coded, with no leave as the reference group. Paths from covariates, factor loadings for PI indicators, and residual covariances between corresponding PI indicators across time were included but are omitted for clarity. Paternity leave duration represents the father’s total paternity leave duration reported at the 6-month follow-up. Arrows represent model-specified associations. Model fit indices: χ^2^(194) = 309.282, χ^2^/df = 1.594, CFI = 0.961, TLI = 0.946, RMSEA = 0.039.

**Figure 3:**
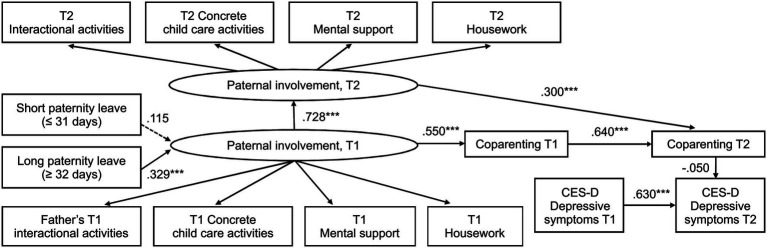
Two-wave structural equation model of modeled associations among paternity leave duration, paternal involvement, coparenting, and maternal depressive symptoms in multiparas. Note. Standardized path coefficients (β) from the freely estimated model are shown. Solid and dashed lines indicate paths whose 95% bias-corrected bootstrap confidence intervals excluded and included zero, respectively. T1, 3 months postpartum; T2, 6 months postpartum; PI, paternal involvement; CES-D, Center for Epidemiologic Studies Depression Scale. Short and long paternity leave were dummy coded, with no leave as the reference group. Paths from covariates, factor loadings for PI indicators, and residual covariances between corresponding PI indicators across time were included but are omitted for clarity. Paternity leave duration represents the father’s total paternity leave duration reported at the 6-month follow-up. Arrows represent model-specified associations. Model fit indices: χ^2^(194) = 309.282, χ^2^/df = 1.594, CFI = 0.961, TLI = 0.946, RMSEA = 0.039.

Figure 2 presents the final multigroup SEM for primiparous mothers and Figure 3 illustrates this for multiparous mothers. In both figures, T1 indicates 3 months postpartum and T2 indicates 6 months postpartum. Model fit was good: χ^2^(194) = 309.282, χ^2^/df = 1.594, CFI = 0.961, TLI = 0.946, and RMSEA = 0.039.

The model explained a modest to substantial proportion of the variance in the endogenous variables. For primiparas, 16.9% of the variance in PI at 3 months was explained, 62.0% in PI at 6 months, 49.0% in CRS at 3 months, 66.5% in CRS at 6 months, and 45.4% in CES-D at 6 months. For multiparas, the corresponding values were 9.9, 53.0, 38.5, 65.9, and 43.6%. The squared multiple correlations for the endogenous variables are shown in Supplementary Table S5.

Table 3 presents the standardized structural path coefficients for the hypothesized associations. Compared with no paternity leave, long paternity leave was positively associated with PI at 3 months in both parity groups (primiparas: *β* = 0.320, 95% BC CI [0.179, 0.447], *p* < 0.001; multiparas: β = 0.329, 95% BC CI [0.152, 0.490], *p* = 0.001). In contrast, short paternity leave did not differ significantly from no paternity leave in its association with PI at 3 months (primiparas: *β* = 0.101, 95% BC CI [−0.029, 0.230], *p* = 0.116; multiparas: *β* = 0.115, 95% BC CI [−0.079, 0.294], *p* = 0.234).

**Table 3:** Standardized structural path coefficients for the hypothesized associations.

Path	Primiparas β	Primiparas 95% BC CI	Primiparas *p*	Multiparas β	Multiparas 95% BC CI	Multiparas *p*
Short paternity leave→PI, 3 months	0.101	[−0.029, 0.230]	0.116	0.115	[−0.079, 0.294]	0.234
Long paternity leave→PI, 3 months	0.320	[0.179, 0.447]	< 0.001	0.329	[0.152, 0.490]	0.001
PI, 3 months→CRS, 3 months	0.656	[0.537, 0.766]	< 0.001	0.550	[0.403, 0.676]	0.001
PI, 3 months→PI, 6 months	0.787	[0.671, 0.866]	0.001	0.728	[0.513, 0.871]	0.001
CRS, 3 months→CRS, 6 months	0.501	[0.358, 0.625]	< 0.001	0.640	[0.509, 0.760]	< 0.001
PI, 6 months→CRS, 6 months	0.426	[0.271, 0.564]	0.001	0.300	[0.121, 0.457]	0.002
CRS, 6 months→CES-D, 6 months	−0.107	[−0.208, −0.017]	0.018	−0.050	[−0.193, 0.084]	0.491
CES-D, 3 months→CES-D, 6 months	0.654	[0.498, 0.769]	0.001	0.630	[0.472, 0.764]	0.001

The table shows standardized coefficients (β), bias-corrected 95% bootstrap confidence intervals (CI), and bootstrap *p* values. PI, paternal involvement in childcare and housework; CRS, Coparenting Relationship Scale; CES-D, Center for Epidemiologic Studies Depression Scale. Bootstrap samples = 5,000.

PI at 3 months was positively associated with CRS at 3 months in both groups (primiparas: *β* = 0.656, 95% BC CI [0.537, 0.766], *p* < 0.001; multiparas: *β* = 0.550, 95% BC CI [0.403, 0.676], *p* = 0.001). PI at 3 months was also strongly associated with PI at 6 months (primiparas: β = 0.787, 95% BC CI [0.671, 0.866], *p* = 0.001; multiparas: *β* = 0.728, 95% BC CI [0.513, 0.871], *p* = 0.001). CRS at 3 months was associated with CRS at 6 months (primiparas: *β* = 0.501, 95% BC CI [0.358, 0.625], *p* < 0.001; multiparas: *β* = 0.640, 95% BC CI [0.509, 0.760], *p* < 0.001), and PI at 6 months was positively associated with CRS at 6 months (primiparas: *β* = 0.426, 95% BC CI [0.271, 0.564], *p* = 0.001; multiparas: *β* = 0.300, 95% BC CI [0.121, 0.457], *p* = 0.002). Regarding maternal depressive symptoms, CRS at 6 months was negatively associated with CES-D at 6 months among primiparas (*β* = −0.107, 95% BC CI [−0.208, −0.017], *p* = 0.018), whereas the corresponding group-specific estimate was not statistically supported among multiparas (*β* = −0.050, 95% BC CI [−0.193, 0.084], *p* = 0.491). CES-D at 3 months was strongly associated with CES-D at 6 months in both groups (primiparas: *β* = 0.654, 95% BC CI [0.498, 0.769], *p* = 0.001; multiparas: *β* = 0.630, 95% BC CI [0.472, 0.764], *p* = 0.001). However, under the metric-invariant model, constraining the unstandardized CRS-at-6-months to CES-D-at-6-months path to equality across parity groups did not significantly worsen model fit, χ^2^(200) = 319.592 versus χ^2^(201) = 320.074, Δχ^2^(1) = 0.482, *p* = 0.488. Thus, a statistically significant between-group difference was not established. Supplementary Table S6 shows the standardized covariate path coefficients. Maternal age and employment were not significantly associated with PI at 3 months, CRS at 3 months, or CES-D at 6 months in either group. By contrast, greater perceived financial adequacy was positively associated with PI at 3 months among primiparas (*β* = 0.284, 95% BC CI [0.154, 0.419], *p* = 0.001) and with CRS at 3 months among multiparas (*β* = 0.229, 95% BC CI [0.072, 0.376], *p* = 0.003). Associations between perceived financial adequacy and CES-D at 6 months were negative but did not reach statistical significance in either group.

### 3.4 Modeled indirect associations

Table 4 presents key standardized modeled indirect associations of paternity leave duration and family-process variables on PI at 6 months, CRS at 3 and 6 months, and CES-D at 6 months. Relative to no paternity leave, long paternity leave was positively associated with PI at 6 months through the modeled pathways in both groups (primiparas: *β* = 0.252, 95% BC CI [0.136, 0.364], *p* < 0.001; multiparas: *β* = 0.239, 95% BC CI [0.107, 0.388], *p* = 0.001). Long paternity leave was also indirectly associated with CRS at 3 months (primiparas: *β* = 0.210, 95% BC CI [0.111, 0.307], *p* < 0.001; multiparas: *β* = 0.181, 95% BC CI [0.083, 0.291], *p* = 0.001) and CRS at 6 months (primiparas: *β* = 0.212, 95% BC CI [0.118, 0.306], *p* < 0.001; multiparas: *β* = 0.187, 95% BC CI [0.084, 0.299], *p* = 0.001).

**Table 4:** Standardized modeled indirect associations in the final multigroup SEM.

Path	Primiparas β	Primiparas 95% BC CI	Primiparas *p*	Multiparas β	Multiparas 95% BC CI	Multiparas *p*
Long paternity leave→PI, 6 months	0.252	[0.136, 0.364]	< 0.001	0.239	[0.107, 0.388]	0.001
Long paternity leave→CRS, 3 months	0.210	[0.111, 0.307]	< 0.001	0.181	[0.083, 0.291]	0.001
Long paternity leave→CRS, 6 months	0.212	[0.118, 0.306]	< 0.001	0.187	[0.084, 0.299]	0.001
Long paternity leave→CES-D, 6 months	−0.023	[−0.048, −0.006]	0.010	−0.009	[−0.043, 0.015]	0.421
PI, 3 months→CRS, 6 months	0.664	[0.568, 0.744]	0.001	0.570	[0.429, 0.696]	0.001
PI, 6 months→CES-D, 6 months	−0.046	[−0.098, −0.008]	0.015	−0.015	[−0.068, 0.025]	0.415
CRS, 3 months→CES-D, 6 months	−0.053	[−0.113, −0.011]	0.013	−0.032	[−0.123, 0.052]	0.479
PI, 3 months→CES-D, 6 months	−0.071	[−0.139, −0.012]	0.017	−0.029	[−0.114, 0.049]	0.486

Total standardized modeled indirect associations are presented with bias-corrected 95% bootstrap confidence intervals (CI) and bootstrap p values. PI, paternal involvement in childcare and housework; CRS, Coparenting Relationship Scale; CES-D, Center for Epidemiologic Studies Depression Scale. Short and long paternity leave were dummy coded, with no paternity leave as the reference group. Bootstrap samples = 5,000.

In the freely estimated structural model, the total indirect association between long paternity leave and CES-D at 6 months was statistically supported among primiparas (β = −0.023, 95% BC CI [−0.048, −0.006], *p* = 0.010) but not among multiparas (*β* = −0.009, 95% BC CI [−0.043, 0.015], *p* = 0.421). Under the metric-invariant model, the corresponding unstandardized total indirect association was B = −0.416, 95% bias-corrected CI [−0.886, −0.104], *p* = 0.009 among primiparas and B = −0.191, 95% bias-corrected CI [−0.822, 0.324], *p* = 0.417 among multiparas. The formal between-group contrast was not statistically significant, ΔB = −0.225, 95% bias-corrected CI [−0.903, 0.441], *p* = 0.454. Thus, the difference between a statistically supported estimate in one group and a nonsignificant estimate in the other should not be interpreted as evidence of a parity difference. Among primiparas, PI at 3 months was also indirectly associated with lower CES-D at 6 months (*β* = −0.071, 95% BC CI [−0.139, −0.012], *p* = 0.017), and CRS at 3 months showed a small indirect association with lower CES-D at 6 months through later CRS (*β* = −0.053, 95% BC CI [−0.113, −0.011], *p* = 0.013); the corresponding estimates were not statistically supported among multiparas. Overall, the results describe model-specified structural and indirect associations. The standardized total indirect association between long leave and CES-D among primiparas was very small (*β* = −0.023), and the corresponding metric-invariant unstandardized estimate was less than one point on the 0–60 CES-D scale (B = −0.416). Its clinical or public-health significance is therefore uncertain.

### 3.5 Verification of the hypotheses

H1 was supported for long paternity leave in both parity groups. Compared with no paternity leave, long paternity leave was associated with higher PI at 3 months, and PI at 3 months was associated with better CRS at 3 months among both primiparas and multiparas. Short paternity leave was not significantly associated with PI at 3 months in either group.

H2 was supported with respect to the 3-to-6-month continuity of PI and CRS in both groups. The CRS-at-6-months to CES-D-at-6-months association was statistically supported within primiparas but not within multiparas; however, the formal between-group test did not establish a significant parity difference. The parity-specific findings should therefore be interpreted as group-specific statistical patterns rather than evidence that the association differed by parity.

## 4 Discussion

### 4.1 Summary of main findings

This study examined modeled associations among paternity leave duration, mother-reported paternal involvement, coparenting, and maternal depressive symptoms across the short interval from 3 to 6 months postpartum. Long leave, compared with no leave, was associated with greater paternal involvement at 3 months in both parity groups, whereas short leave was not clearly associated with paternal involvement. Paternal involvement and coparenting showed continuity from 3 to 6 months. Within-group estimates for the CRS-at-6-months to CES-D-at-6-months association and for the total indirect association between long leave and CES-D were statistically supported among primiparas but not among multiparas. However, formal metric-invariant comparisons did not establish significant between-group differences for either association. The results therefore identify group-specific patterns within the specified model rather than a pathway unique to or stronger among primiparas. The modeled indirect association with depressive symptoms was very small. These findings describe short-term observational patterns; causal interpretation and clinical significance require further study.

### 4.2 Paternity leave and paternal involvement

The association between longer leave and greater mother-reported paternal involvement is broadly consistent with earlier observational work (12, 13, 29, 30) and with Japanese evidence that work-related time can constrain fathers’ engagement in childcare (10, 11). This pattern may help explain why findings on paternity leave and parental mental health have been mixed: leave duration alone may be less informative than whether leave is accompanied by substantive participation in childcare, housework, and emotional support. However, leave duration was not randomly assigned. Fathers who took longer leave may have differed in pre-existing involvement, gender-role attitudes, relationship quality, workplace flexibility, job security, income, or mental health. The present association therefore should not be interpreted as evidence that extending leave would cause greater paternal involvement or better maternal mental health.

### 4.3 Coparenting continuity during the early postpartum period

The data also indicate that coparenting appeared to show continuity from 3 to 6 months postpartum, consistent with work describing coparenting as a family-level process across the first 2 years (16). Feinberg’s (17) account of coparenting—how adults managing parenthood together coordinate, support, and sometimes undermine each other—is explicitly distinct from the couple’s general relationship quality; this distinction is relevant here. In the present model, paternal involvement at 3 months was associated with coparenting at that time, and both paternal involvement and coparenting exhibited continuity to 6 months. These patterns are consistent with, but do not demonstrate, the possibility that paternal involvement and cooperative coparenting are linked during the early postpartum period. Because PI and CRS were measured contemporaneously at 3 months and the model included only two waves, their temporal and causal direction cannot be established.

### 4.4 Parity-specific patterns and broader context

The group-specific estimates were statistically supported among primiparas but not among multiparas for the later CRS-to-CES-D association and for the total indirect association from long leave to CES-D. However, the formal between-group tests were nonsignificant. The findings therefore do not establish that these associations were stronger among primiparas or absent among multiparas. The smaller multiparous sample (*n* = 152), including only 41 families in the long-leave category, may also have reduced precision and power for detecting small indirect associations. Parity may still provide a useful context for generating hypotheses because first-time families are establishing parenting and coparenting roles, whereas multiparous families may face older-child care and more complex household demands. Evidence that dyadic coparenting interactions during the transition to parenthood are associated with subsequent postpartum depressive symptoms further supports examining these family processes during this period (31). Nevertheless, the present parity-specific patterns should be interpreted as exploratory.

### 4.5 Policy and perinatal practice implications

The findings may inform hypotheses about family processes accompanying paternity leave. Given the very small modeled indirect association with CES-D, the results do not establish a clinically meaningful maternal-health benefit. Perinatal services may consider supporting couples in developing concrete caregiving, housework, emotional-support, and coparenting routines during the postpartum period (32–36). Such support could be evaluated in future intervention studies.

### 4.6 Limitations and future directions

Several limitations should be recognized. First, PI and CRS were measured contemporaneously at 3 months, and CRS and CES-D were measured contemporaneously at 6 months. Adjustment for CES-D at 3 months accounts for symptom stability but does not establish directionality. Maternal psychological distress may also have been associated with fathers’ leave-taking decisions. Reciprocal associations and shared unobserved family functioning therefore remain plausible, and the two-wave observational design cannot establish causal direction or persistence beyond 6 months.

Second, all principal variables were reported by mothers. Maternal perceptions are relevant to maternal adjustment, but they are not independent assessments of fathers’ behavior or coparenting. Common-source and mood-congruent reporting bias may have inflated associations because mothers with more depressive symptoms may have rated PI or coparenting less favorably. Third, selection into paternity leave and residual confounding cannot be ruled out. Unmeasured factors such as gender-role attitudes, pre-existing paternal involvement, relationship quality, workplace flexibility, job security, income, paternal mental health, and informal support may have influenced both leave-taking and family outcomes. Infant health, temperament, sleep, feeding difficulties, and caregiving demands were also unavailable and may be important omitted confounders.

Fourth, 159 of 555 baseline participants were not included in the final analyses. Although most assessed baseline characteristics were comparable, included participants had slightly higher baseline CRS scores (Cohen’s d = 0.24), so attrition bias cannot be excluded. Fifth, no *a priori* power analysis was conducted for parity-specific indirect associations. The multiparous sample was smaller (*n* = 152), with 41 families in the long-leave category, and confidence intervals were correspondingly imprecise. Sixth, the short-leave category was heterogeneous, and the latent PI construct does not identify which individual domain was most influential. Future studies should use at least three waves, obtain reports from both parents and observational measures, include infant and workplace characteristics, and evaluate clinical relevance in adequately powered causal or intervention designs.

## 5 Conclusion

Long paternity leave, relative to no leave, was associated with greater mother-reported paternal involvement at 3 months, and paternal involvement and coparenting showed short-term continuity from 3 to 6 months. The modeled indirect association between long leave and maternal depressive symptoms was very small and statistically supported among primiparas but not among multiparas; however, formal comparisons did not establish a parity difference. These observational findings identify short-term family-process associations that warrant further investigation.

## Data Availability

The datasets presented in this article are not readily available because they contain sensitive personal information. Requests to access the datasets should be directed to the corresponding author.
