# Supplementary material for: Paternity leave duration and maternal depressive symptoms: a longitudinal study of paternal involvement and coparenting in Japan
Source: Front Public Health. 2026 Jul 22;14:1891071. doi: 10.3389/fpubh.2026.1891071 (PMC13437384; doi:10.3389/fpubh.2026.1891071)
Supplement: Supplementary file 1 [file Data_sheet_1.pdf]

Supplementary Material

1 Supplementary Tables

Table S1. Baseline characteristics of participants included and not included in the final longitudinal analyses

Baseline characteristic	Included in final analysis	Not included	p value	Effect size
	(n = 396)	(n = 159)		
Maternal age, years	33.03 ± 4.72	32.81 ± 4.52	0.615	d = .047
Paternal age, years	34.68 ± 5.97	34.32 ± 5.86	0.521	d = .060
**Parity, n (%)**			**0.173**	**V = .058**
Primiparous	244 (61.6)	88 (55.3)		
Multiparous	152 (38.4)	71 (44.7)		
Perceived financial adequacy	2.62 ± 0.76	2.64 ± 0.81	0.847	d = −.018
**Maternal employment, n (%)**			**0.828**	**V = .009**
Employed	290 (73.2)	115 (72.3)		
Not employed	106 (26.8)	44 (27.7)		
**Paternity leave duration reported at T1, n (%)**			**0.432**	**V = .055**
No leave	174 (43.9)	78 (49.1)		
Short leave (1–31 days)	95 (24.0)	31 (19.5)		
Long leave (≥32 days)	127 (32.1)	50 (31.4)		
PI: Interactional activities, T1	3.11 ± 0.58	3.11 ± 0.55	0.893	d = .013
PI: Concrete childcare activities, T1	2.85 ± 0.74	2.72 ± 0.67	0.071	d = .170
PI: Mental support, T1	2.96 ± 0.74	2.92 ± 0.63	0.622	d = .046
PI: Housework, T1	2.60 ± 0.77	2.54 ± 0.80	0.392	d = .080
CES-D total score, T1	11.51 ± 9.08	12.16 ± 9.15	0.446	d = −.072
CRS total score, T1	3.01 ± 0.64	2.85 ± 0.64	0.01	d = .244

Note. Values are presented as mean ± standard deviation or *n* (%). Between-group differences were examined using independent-samples *t* tests for continuous variables and Pearson's chi-square tests for categorical variables. Effect sizes are reported as Cohen's *d* for continuous variables and Cramer's *V* for categorical variables. Positive Cohen's *d* values indicate higher mean values among participants included in the

final analysis. Paternity leave duration reported at T1 was used for the attrition analysis because 6-month paternity-leave data were unavailable for participants without usable follow-up data. T1 = 3 months postpartum; PI = paternal involvement; CRS = Coparenting Relationship Scale; CES-D = Center for Epidemiologic Studies Depression Scale.

Table S2. Internal consistency reliability at 6 months postpartum and number of items for the study measures

Scale	Subscale	α	Number of Items
Child care activities and housework	Interactional Activities	0.768	5
	Concrete Child Care Activities	0.839	6
	Mental Support	0.758	4
	Housework	0.733	4
CRS	CRS Total Score	0.85	35
	Agreement	0.798	4
	Closeness	0.777	5
	Exposure to conflict	0.886	5
	Support	0.902	6
	Undermining	0.846	6
	Endorsement of partner's parenting	0.851	7
	Division of labor	0.633	2
CES-D	Depressive Symptoms Total Score	0.866	20

Note. CRS = Coparenting Relationship Scale; CES-D = Center for Epidemiologic Studies Depression Scale.

Table S3. Bivariate correlations among study variables in primiparas (n = 244)

No.	Variable	1	2	3	4	5	6	7	8	9	10	11	12	13	14	15	16
1	Maternal age	-															
2	Maternal employment	-0.146*	-														
3	Perceived financial adequacy	0.159*	-0.128*	-													
4	Short paternity leave	0.003	-0.136*	0.028	-												
5	Long paternity leave	-0.148*	-0.044	-0.039	-0.364**	-											
6	PI1, 3 months	-0.024	-0.057	0.218**	-0.040	0.252**	-										
7	PI2, 3 months	-0.067	0.040	0.199**	-0.045	0.252**	0.655**	-									
8	PI3, 3 months	-0.120	-0.004	0.173**	-0.005	0.184**	0.618**	0.601**	-								
9	PI4, 3 months	-0.017	-0.084	0.181**	-0.034	0.206**	0.474**	0.541**	0.562**	-							

10	CRS, 3 months	-0.073	-0.039	0.285**	0.051	0.200**	0.494**	0.466**	0.629**	0.460**	-							
11	PI1, 6 months	-0.027	-0.011	0.193**	0.027	0.180**	0.606**	0.512**	0.495**	0.421**	0.452**	-						
12	PI2, 6 months	-0.114	0.106	0.148*	-0.014	0.184**	0.452**	0.623**	0.468**	0.456**	0.418**	0.622**	-					
13	PI3, 6 months	-0.114	0.009	0.155*	0.006	0.202**	0.442**	0.432**	0.719**	0.419**	0.585**	0.574**	0.576**	-				
14	PI4, 6 months	-0.082	0.007	0.144*	-0.060	0.179**	0.384**	0.381**	0.428**	0.718**	0.414**	0.517**	0.561**	0.522**	-			
15	CRS, 6 months	-0.094	-0.036	0.284**	0.088	0.155*	0.423**	0.434**	0.602**	0.469**	0.749**	0.506**	0.540**	0.673**	0.466**	-		
16	CES-D, 3 months	-0.047	-0.008	-0.274**	-0.091	0.021	-0.130*	-0.091	-0.238**	-0.077	-0.155*	-0.160*	0.015	-0.182**	-0.048	-0.151*	-	
17	CES-D, 6 months	-0.025	0.023	-0.301**	-0.179**	0.094	-0.066	-0.028	-0.154*	-0.002	-0.119	-0.147*	-0.015	-0.193**	-0.002	-0.230**	0.677**	

Note. Pearson’s correlation coefficients are shown below the diagonal. PI = paternal involvement in childcare and housework; PI1 = interactional activities; PI2 = concrete child care activities; PI3 = mental support; PI4 = housework; CRS = Coparenting Relationship Scale; CES-D = Center for Epidemiologic Studies Depression Scale. Maternal employment, short paternity leave, and long paternity leave were coded as binary variables (0/1). Short and long paternity leave were dummy coded, with no paternity leave as the reference group. * p < 0 .05. ** p < 0.01.

Table S4. Bivariate correlations among study variables in multiparas (n = 152)

No.	Variable	1	2	3	4	5	6	7	8	9	10	11	12	13	14	15	16
1	Maternal age	-															
2	Maternal employment	0.032	-														
3	Perceived financial adequacy	0.091	-0.122	-													
4	Short paternity leave	0.055	0.024	0.126	-												
5	Long paternity leave	-0.086	-0.052	-0.009	-0.388**	-											
6	PI1, 3 months	0.037	-0.023	0.019	-0.017	0.238**	-										
7	PI2, 3 months	0.048	0.014	0.09	-0.014	0.254**	0.619**	-									
8	PI3, 3 months	0.003	-0.072	0.11	0.024	0.181*	0.572**	0.694**	-								
9	PI4, 3 months	0.009	-0.037	-0.024	-0.055	0.267**	0.445**	0.584**	0.567**	-							

10	CRS, 3 months	0.024	-0.108	0.283**	0.030	0.194*	0.304**	0.453**	0.603**	0.315**	-							
11	PI1, 6 months	-0.002	0.023	0.060	-0.038	0.218**	0.580**	0.470**	0.486**	0.329**	0.251**	-						
12	PI2, 6 months	-0.007	0.104	0.042	-0.061	0.282**	0.422**	0.606**	0.438**	0.411**	0.296**	0.676**	-					
13	PI3, 6 months	-0.077	-0.04	0.111	-0.065	0.229**	0.449**	0.424**	0.731**	0.380**	0.483**	0.596**	0.531**	-				
14	PI4, 6 months	-0.032	-0.004	-0.004	-0.075	0.279**	0.305**	0.336**	0.401**	0.652**	0.192*	0.375**	0.465**	0.466**	-			
15	CRS, 6 months	-0.047	0.043	0.189*	0.012	0.169*	0.271**	0.445**	0.633**	0.376**	0.765**	0.366**	0.411**	0.594**	0.292**	-		
16	CES-D, 3 months	0.044	-0.008	-0.186*	-0.045	-0.085	-0.201*	-0.185*	-0.258**	-0.215**	-0.210**	-0.141	-0.075	-0.205*	-0.027	-0.236**	-	
17	CES-D, 6 months	-0.001	0.107	-0.273**	-0.130	-0.062	-0.117	-0.036	-0.217**	-0.111	-0.204*	-0.176*	-0.017	-0.237**	0.014	-0.215**	0.652**	

Note. Pearson's correlation coefficients are shown below the diagonal. PI = paternal involvement in childcare and housework; PI1 = interactional activities; PI2 = concrete child care activities; PI3 = mental support; PI4 = housework; CRS = Coparenting Relationship Scale; CES-D = Center for Epidemiologic Studies Depression Scale. Maternal employment, short paternity leave, and long paternity leave were coded as binary variables (0/1). Short and long paternity leave were dummy coded, with no paternity leave as the reference group. * $p < 0.05$. ** $p < 0.01$.

Table S5. Squared multiple correlations (R^2) for endogenous variables

Endogenous variable	Primiparas R^2	Multiparas R^2
PI, 3 months	0.169	0.099
PI, 6 months	0.620	0.530
CRS, 3 months	0.490	0.385
CRS, 6 months	0.665	0.659
CES-D, 6 months	0.454	0.436

Note. Values indicate the proportion of variance explained for each endogenous variable in each group. PI = paternal involvement in childcare and housework; CRS = Coparenting Relationship Scale; CES-D = Center for Epidemiologic Studies Depression Scale.

Table S6. Standardized covariate path coefficients

Path	Primiparas β	Primiparas 95% BC CI	Primiparas p	Multiparas β	Multiparas 95% BC CI	Multiparas p
PI, 3 months Maternal age	-0.083	[-0.221, 0.062]	0.275	0.049	[-0.111, 0.210]	0.515
PI, 3 months Maternal employment	0.03	[-0.109, 0.171]	0.67	0	[-0.192, 0.181]	0.968
PI, 3 months Perceived financial adequacy	0.284	[0.154, 0.419]	0.001	0.069	[-0.124, 0.266]	0.479
CRS, 3 months Maternal age	-0.035	[-0.146, 0.080]	0.552	-0.013	[-0.140, 0.108]	0.797
CRS, 3 months Maternal employment	-0.014	[-0.124, 0.096]	0.772	-0.068	[-0.193, 0.060]	0.312
CRS, 3 months Perceived financial adequacy	0.12	[-0.003, 0.233]	0.057	0.229	[0.072, 0.376]	0.003
CES-D, 6 months Maternal age	0.013	[-0.076, 0.113]	0.714	-0.022	[-0.157, 0.110]	0.757
CES-D, 6 months Maternal employment	0.014	[-0.106, 0.127]	0.874	0.1	[-0.023, 0.238]	0.109
CES-D, 6 months Perceived financial adequacy	-0.1	[-0.233, 0.012]	0.084	-0.138	[-0.283, 0.003]	0.057

Note. Table shows standardized coefficients (β), bias-corrected 95% bootstrap confidence intervals (CI), and bootstrap p values. PI = paternal involvement; CRS = Coparenting Relationship Scale; CES-D = Center for Epidemiologic Studies Depression Scale.

Table S7. Standardized factor loadings, latent-factor correlations, and measurement invariance of paternal involvement across parity groups

Panel A. Standardized factor loadings and latent-factor correlations from the configural model

Time / parameter	Indicator	Primiparas (n = 244)	Multiparas (n = 152)
3 months (T1)	Interactional activities	0.775	0.697
	Concrete childcare activities	0.809	0.878
	Mental support	0.781	0.795
	Housework	0.676	0.658
6 months (T2)	Interactional activities	0.782	0.803
	Concrete childcare activities	0.797	0.825
	Mental support	0.733	0.691
	Housework	0.676	0.541
Latent-factor correlation	PI at T1 with PI at T2	0.773	0.715

Panel B. Model fit for the configural and metric-invariance models

Model	χ^2	df	p	χ^2/df	CFI	TLI	RMSEA (90% CI)	PCLOSE
Configural	33.849	30	0.287	1.128	0.998	0.996	.018 [.000, .044]	0.986
Metric invariance	44.467	36	0.157	1.235	0.996	0.993	.024 [.000, .046]	0.98

Panel C. Change in fit for the metric-invariance model relative to the configural model

Comparison	$\Delta\chi^2$	Δdf	p for $\Delta\chi^2$	ΔCFI	ΔRMSEA
Metric invariance vs. configural	10.618	6	0.101	−.002	0.006
